# A chromosome 4 trisomy contributes to increased fluconazole resistance in a clinical isolate of *Candida albicans*

**DOI:** 10.1099/mic.0.000478

**Published:** 2017-06-22

**Authors:** Matthew Z. Anderson, Amrita Saha, Abid Haseeb, Richard J. Bennett

**Affiliations:** Department of Molecular Microbiology and Immunology, Brown University, Providence, RI, 02912, USA

**Keywords:** *Candida*, drug resistance, aneuploidy, fluconazole, efflux pumps

## Abstract

*Candida albicans* is an important opportunistic fungal pathogen capable of causing both mucosal and disseminated disease. Infections are often treated with fluconazole, a front-line antifungal drug that targets the biosynthesis of ergosterol, a major component of the fungal cell membrane. Resistance to fluconazole can arise through a variety of mechanisms, including gain-of-function mutations, loss of heterozygosity events and aneuploidy. The clinical isolate P60002 was found to be highly resistant to azole-class drugs, yet lacked mutations or chromosomal rearrangements known to be associated with azole resistance. Transcription profiling suggested that increased expression of two putative drug efflux pumps, *CDR11* and *QDR1*, might confer azole resistance. However, ectopic expression of the P60002 alleles of these genes in a drug-susceptible strain did not increase fluconazole resistance. We next examined whether the presence of three copies of chromosome 4 (Chr4) or chromosome 6 (Chr6) contributed to azole resistance in P60002. We established that Chr4 trisomy contributes significantly to fluconazole resistance, whereas Chr6 trisomy has no discernible effect on resistance. In contrast, a Chr4 trisomy did not increase fluconazole resistance when present in the standard SC5314 strain background. These results establish a link between Chr4 trisomy and elevated fluconazole resistance, and demonstrate the impact of genetic background on drug resistance phenotypes in *C. albicans*.

## Abbreviations

ABC, ATP-binding cassette; MDR, multidrug resistance; MFS, major facilitator superfamily; qRT-PCR, quantitative reverse-transcription PCR; PDR, pleiotropic drug resistance; UTR, untranslated region.

## Introduction

Mechanisms for generating resistance to stress are critical for the survival of microbial species. The fungus *Candida albicans* resides as a benign commensal in most humans, but overgrowth of the natural host niche is capable of producing both superficial and life-threatening infections [1, 2]. Symptomatic infections are especially prevalent in immunocompromised patients and those undergoing organ transplant or chemotherapy, and during heavy steroid or antibiotic use [3, 4]. Four classes of antifungal drugs are generally used in the preventative care and treatment of *C. albicans* infections: azoles, polyenes, allylamines and echinocandins [5]. The triazole drug fluconazole is frequently used in the clinic due to its low cost, efficacy, lack of toxicity and ease of administration [6]. Consequently, administration of prophylactic fluconazole in cases of immune suppression and in neonates has become widespread [7]. Fluconazole acts to block activity of lanosterol 14α-demethylase (the product of the *ERG11* gene) leading to ergosterol depletion and accumulation of toxic sterol byproducts in the cell membrane [8]. These changes cause reduced membrane fluidity and increased membrane leakage, and ultimately inhibit fungal cell growth and division [9, 10].

Reports of resistance to fluconazole have increased due to more frequent use of immunosuppression and antifungal prophylaxis [11, 12]. The fungistatic nature of fluconazole may also facilitate the emergence of resistance during prolonged treatment. Common mechanisms of fluconazole resistance fall into three categories: (1) genetic alterations to the Erg11 drug target, (2) compensatory changes in ergosterol biosynthesis and (3) reduced effective drug concentrations inside the cell. More specifically, point mutations in *ERG11* have been identified that reduce or abolish the binding capacity of fluconazole to its target protein [13, 14]. Alternatively, increased expression of the *ERG11* gene can occur due to increased gene dosage or due to *trans*-acting mutations, thereby reducing drug efficacy [15, 16]. For example, a zinc cluster transcription factor, Upc2, activates *ERG11* gene expression and hyperactive alleles of Upc2 have been identified in fluconazole-resistant isolates [17, 18]. An alternative metabolic mechanism of resistance occurs when loss-of-function mutations in *ERG3* prevent the accumulated 14α-methyl sterol from being converted into toxic 3,6-diol derivatives [19]. Finally, increased activity of drug transporters can deplete the intracellular accumulation of fluconazole to promote resistance. For example, the ABC transporters encoded by Cdr1 and Cdr2, as well as the major facilitator superfamily (MFS) drug efflux pump encoded by Mdr1, can reduce fluconazole concentrations by active efflux [20, 21]. Hyperactive alleles of the Mrr1, Mrr2, or Tac1 transcription factors also increase drug efflux through upregulation of *CDR1* and *CDR2* genes [22, 23].

Fluconazole resistance often arises through multiple mechanisms and even single mutations can impact resistance through pleiotropic effects on multiple gene classes. For example, hyperactive Upc2 can directly upregulate expression of both *ERG3* and *ERG11,* as well as the drug efflux pumps *CDR1* and *MDR1* [24]. In the clinic, drug resistance often develops through the progressive accumulation of multiple independent mutations that cause incremental increases in resistance. Each mutation confers additive resistance that results in full protection when present in combination [25–27].

Large-scale genomic changes such as chromosomal rearrangements and aneuploidy can also drive the emergence of fluconazole resistance. In particular, formation of an isochromosome comprising the two left arms of Chr5 (i5L) confers high levels of resistance to azole drugs in *C. albicans* [15]. The left arm of Chr5 encodes both the fluconazole target Erg11 and the transcription factor Tac1. Elevated expression of these genes due to increased copy number in strains carrying i5L mediates increased drug resistance [28]. Changes in the complement of chromosomes have also been associated with drug resistance in other fungal pathogens [29]. In general, the emergence of aneuploid forms provides a way for cells to rapidly generate genotypic and phenotypic diversity without permanently committing to the mutant genotype [30–32]. This rapid but imperfect mechanism of adaptation can subsequently be replaced by more refined adaptive changes that have a lower fitness cost [33].

In some cases, clinical infections with *C. albicans* persist even when azole drugs are used at concentrations well above the minimum inhibitory concentration (MIC) [34]. In these examples, cell subpopulations continue to grow above their MIC in a phenomenon known as tolerance or heteroresistance [35]. Robust production of β-glucan and extracellular DNA during biofilm formation promotes tolerance to multiple antifungal agents in *C. albicans* [36]. Defects in intrinsic cellular function such as the calcineurin pathway, Hsp90, and membrane trafficking through endosomes can also contribute to azole tolerance [37–39]. Mutations leading to elevated azole tolerance may commonly precede progression to full drug resistance [40].

The prevalence of fluconazole resistance in clinical *C. albicans* isolates can vary significantly, with reports of resistance in 2–25 % of isolates [41–43]. While sequencing can identify causative mutations in known resistance loci, many resistant isolates lack any established signatures of drug resistance [44]. In particular, it is possible that aneuploid forms other than i5L contribute to azole resistance in *C. albicans*. Multiple chromosomal aneuploidies have been observed in fluconazole-resistant isolates in addition to i5L, but a causal relationship between many of these structural changes and resistance has not been established [15, 45–47]. Chromosomal changes may be a direct consequence of fluconazole actively inducing the formation of aneuploid cells through disruption of the cell cycle [48]. Extensive karyotypic variation is commonly observed in *C. albicans* isolates [49–51] and reveals that cells can adopt a range of ploidy states from haploid to tetraploid, as well as multiple aneuploid variants [47, 52, 53]. It is therefore an important question to determine which chromosomal aneuploidies are simply a consequence of drug-induced genomic instability, and which provide a selective advantage in the presence of the drug.

Here, we investigated drug resistance in clinical isolate P60002, which lacks any established signatures of fluconazole resistance [54]. Two putative efflux pumps, *CDR11* and *QDR1*, exhibited significantly elevated transcript levels in P60002 compared to fluconazole-susceptible strains. However, ectopic expression of the corresponding P60002 alleles in a susceptible isolate did not significantly alter fluconazole resistance. Instead, we demonstrated that drug resistance in P60002 is due, at least in part, to the presence of a Chr4 trisomy. Loss of the trisomic chromosome significantly reduced fluconazole resistance, although the euploid derivative still exhibited clinically significant levels of resistance. In contrast, derivatives of SC5314 that carried a Chr4 trisomy did not show elevated fluconazole resistance. Our results therefore establish that the presence of a trisomic Chr4 contributes to fluconazole resistance in P60002, but does so in a strain-background-dependent manner.

## Methods

### Passaging of *C. albicans* strain P60002

Ninety-six individual colonies of strain P60002 were cultured in liquid medium by serial passaging for 18 days. Cells were cultured in 900 µl yeast extract, peptone and dextrose (YPD) medium at 30 °C on a shaking platform. Every 24 h, 15 µl was transferred to 885 µl of fresh YPD medium.

### Strain construction

The strains are listed in Table S1 (available in the online Supplementary Material). Transformations were performed using lithium acetate as previously described [55]. To integrate *CDR11* and *QDR1* alleles from P60002 into SC5314 (including 5′ UTR, promoter, coding sequence and 3′ UTR), both genes were cloned by PCR from P60002 genomic DNA into pSFS2a [56] using NotI and SacII restriction sites using the primers listed in Table S2. These plasmids were linearized using NotI for *CDR11* or SacI for *QDR1* prior to transformation. Integration at the endogenous *CDR11* or *QDR1* loci was confirmed by PCR using the primers listed in Table S2. Tests for multiple integration events were performed using primers flanking the restriction site used for linearization, but they failed to produce a band, suggesting that single copies of the plasmids integrated into each strain.

### Fluconazole disk diffusion assay

Cells for each strain (including different karyotype configurations) were cultured overnight in YPD. Optical density measurements were used to dilute the cultures to 0.04 OD ml^−1^ (800 000 cells ml^−1^) and 70 µl was plated onto solid YPD agar. Inoculated plates were left for 1 h to dry and a single 25 µg fluconazole disc (Liofilchem, TE, Italy) was placed in the centre of the plate. Cells were allowed to grow for 48 h at 30 °C and images were taken using a Nikon SX40HS Powershot (Nikon, Tokyo, Japan). Drug resistance was quantified using the diskImageR program, which allows the analysis of drug-response parameters [57]. The ‘resistance score’ was calculated as: ((max score−RAD20)/max score)+(0.5*(1-(max score−RAD20)/max score)*FoG80), where RAD20 corresponds to the point where a 20 % reduction in growth occurs, and FoG80 corresponds to the fraction of growth achieved within the zone where there is 80 % growth inhibition [57]. Combining the RAD20 and FoG80 terms provided a measurement of overall drug resistance.

### Broth microdilution assay

Susceptibility to fluconazole using the broth microdilution assay utilized previously published protocols by the National Committee for Clinical Laboratory Standards [58]. Briefly, serial dilutions of fluconazole (from 256 to 0.25 µg ml^−1^) were prepared in RPMI 1640 broth (Life Technologies, Carlsbad, CA, USA) and transferred to 96-well plates, with wells without drug serving as controls. Cells from saturated cultures grown in liquid YPD medium were diluted to an initial OD of 0.03 ml^–1^ in each well and allowed to grow for 24 h. Each strain was tested in biological triplicates with technical duplicates.

### Quantitative reverse-transcription PCR (qRT-PCR)

RNA was prepared using the MasterPure yeast RNA extraction kit (Epicentre, Madison, WI, USA) and cDNA was synthesized using Superscript IV reverse transcriptase with a d(T)_18_ oligonucleotide primer (Thermo Fischer, Waltham, MA, USA). Transcript abundance was quantified by real-time PCR (qRT-PCR) with SYBR green incorporation using a Lightcycler 96 (Roche, Mannheim, Germany). Absolute quantification using the second derivative maximum value was used to calculate the threshold cycle (ΔCT) for each gene using *ACT1* as a control. The primers are listed in Table S2. All qRT-PCR results represent the average abundance of at least three independent experiments per strain.

### ddRAD-Seq analysis of chromosomal copy number

DNA was isolated from 56 passaged P60002 isolates using a MasterPure yeast DNA purification kit (Epicentre, Madison, WI, USA) according to the manufacturer’s instructions. The resulting DNA was suspended in 35 µl of TE (Tris, pH 8.0, EDTA) and stored at 4 °C for library preparation. Isolate DNA was prepared for double-digest restriction-site-associated DNA sequencing using the protocol described in [59]. Briefly, DNA from each sample was digested with BamHI and NdeI restriction enzymes to produce non-complementary overhangs. P1 and P2 adapters that were complementary to each of the overhangs were ligated to the DNA fragments. The P1 adapters encoded unique five-basepair (bp) barcodes and the P2 adapters corresponded to unique six bp barcodes. Adapter-ligated pools of eight samples each were gel-excised between 350–450 bp to limit the DNA library to fragments within that size range. These resulting pools were subsequently purified and amplified using universal Illumina P5 and P7 amplification primers. The prepared libraries were sequenced at the Missouri Genome Sequencing Facility.

The read quality of the sequenced libraries was assessed using FastQC [60]. Reads were demultiplexed using scripts available from the Quantitative Insights into Microbial Ecology (QIIME) consortium. Reads from each sample were individually aligned to the P60002 reference genome [54] using Bowtie2 [61]. Read counts and chromosomal copy numbers were inferred from previously developed custom scripts [62] that were adapted to *C. albicans*. Seven of the 56 samples were removed from analysis due to low read counts or aberrant rDNA overrepresentation and 49 isolates were used for further analysis.

### Growth assays

P60002 isolates that were trisomic for Chr4 and Chr6 (CAY7401, CAY704), trisomic for Chr4 (CAY7403, CAY7404, CAY7405), or disomic for both chromosomes (CAY7417, CAY7418, CAY7420) were grown at 30 °C in liquid YPD or synthetic complete media containing 2% dextrose (SCD) medium overnight. The cultures were then diluted 1 : 200 into fresh YPD or SCD medium. Optical density was measured every 15 min for 48 h with a plate reader (Tecan, Mannedorf, Switzerland) and the polynomial measurement of the curve used to derive relative doubling times.

### Rhodamine 123 accumulation assay

P60002 isolates that were trisomic for Chr4 and Chr6 (CAY7401, CAY704), trisomic for Chr4 (CAY7403, CAY7404, CAY7405), or disomic for both chromosomes (CAY7417, CAY7418, CAY7420) were grown in YPD medium to mid-logarithmic phase. We pelleted 10^7^ cells and washed them twice with phosphate-buffered saline (PBS). The cells were then resuspended in PBS supplemented with 10 mM glucose and grown at 37 °C in the presence of 13 µM rhodamine 123 (Rh123) for 30 min. Cells were washed twice with PBS and OD and the fluorescence was measured using a Qubit 3.0 fluorimeter (ThermoFisher Scientific, Waltham, MA, USA). The dynamic range was assayed by either not adding Rh123 during the incubation phase or not washing the cells in PBS following incubation. This produced a range from 110 to 900 000 relative fluorescent units. Twelve biological replicates were performed for each strain.

## Results

**Fig. 1.**
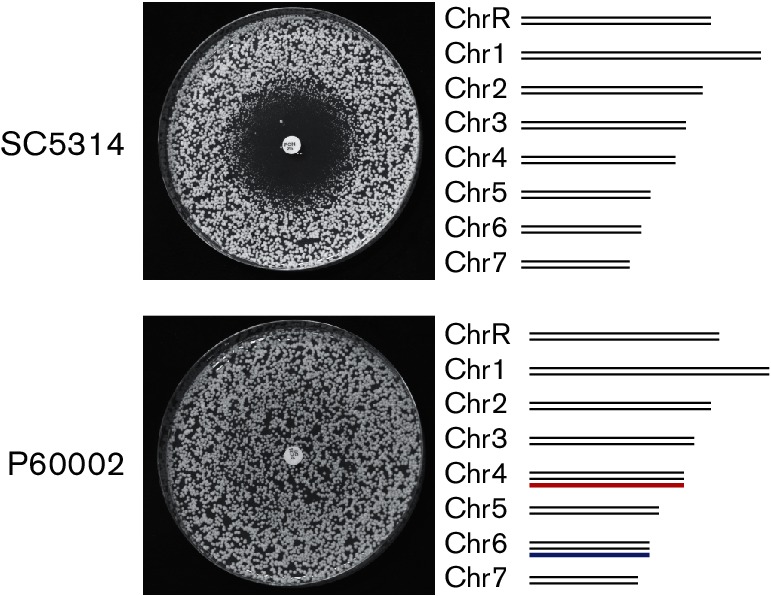
Fluconazole resistance in clinical isolate P60002. *C. albicans* strains SC5314 and P60002 were plated onto YPD medium and allowed to grow in the presence of a 25 µg fluconazole-containing disc. Both strains are diploid, but whereas SC5314 is euploid, P60002 is trisomic for Chr4 (red) and Chr6 (blue).

Previous characterization of a set of 21 sequenced *C. albicans* isolates identified two strains, P94015 and P60002, to be resistant to fluconazole (MIC >256 µg ml^−1^) [54]. P94015 encoded known resistance mutations in both *ERG11,* encoding the molecular target of fluconazole, and *TAC1*, encoding the major transcription factor controlling expression of the drug efflux pump genes *CDR1* and *CDR2* [54]. However, interrogation of the P60002 genome sequence did not reveal any mutations associated with drug resistance, yet this isolate was highly resistant to azoles compared to other *C. albicans* strains, including the standard laboratory strain, SC5314 (Fig. 1). The majority of *C. albicans* isolates are diploid [54], yet the sequenced isolate of P60002 was trisomic for two chromosomes, Chr4 and Chr6, which we investigated further, as outlined below.

### Increased expression of drug transporters in P60002

**Fig. 2.**
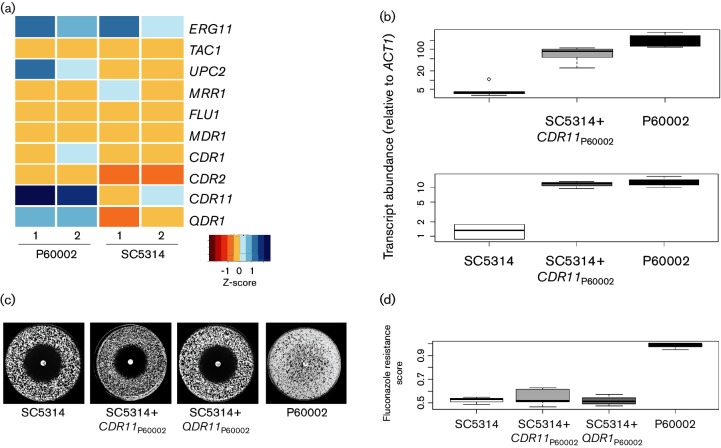
Analysis of the role of *CDR11* and *QDR1*, encoding putative drug efflux pumps, in fluconazole resistance. (a) RNA-Seq expression of select SC5314 and P60002 transcripts. P60002 overexpressed three genes implicated in fluconazole resistance relative to SC5314: *UPC2*, *CDR11* and *QDR1*. (b) Ectopic expression of the P60002 alleles of *CDR11* and *QDR1* in SC5314 increased transcript levels to a similar extent to those present in P60002. (c) Analysis of drug resistance in SC5314 strains ectopically expressing P60002 alleles of *CDR11* or *QDR1.* Cells were plated onto YPD and allowed to grow in the presence of a 25 µg fluconazole disc. Plates were photographed after 2 days. (d) Quantitative analysis of fluconazole resistance in SC5314, P60002 or SC5314-derived strains expressing P60002 alleles of *CDR11* and *QDR1*.

To identify gene expression changes that may contribute to fluconazole resistance, RNA-Seq was previously used to compare transcript abundance between P60002 and SC5314 [54]. Analysis of these data revealed expression differences in three genes with known or putative roles in fluconazole resistance. Specifically, P60002 expressed two putative drug transporters, *CDR11* and *QDR1*, at a significantly higher level than SC5314 (Fig. 2a), and also overexpressed *UPC2*, encoding a major transcriptional regulator of ergosterol synthesis. *CDR11,* located on Chr3, belongs to the pleiotropic drug resistance (PDR) subfamily of ATP-binding cassette (ABC) transporters, whereas *QDR1* on ChrR encodes a major facilitator superfamily drug efflux pump. We note that elevated expression of *UPC2* (located on Chr1) did not lead to a corresponding increase in expression of its target genes in the ergosterol biosynthetic pathway (Fig. 2a), and so the role of this gene was not further investigated.

To determine if the P60002 alleles of *CDR11* or *QDR1* contributed to increased fluconazole resistance, allelic swaps of these loci from P60002 to the azole-susceptible SC5314 strain were performed. Multiple SNPs differentiate the P60002 and SC5314 alleles of these two genes; 48 and 8 SNPs exist in *CDR11* (1 per 94 nt) and *QDR1* (1 per 191 nt), respectively (Tables S3, S4). Each gene, including the predicted promoter and untranslated regions (UTRs), was cloned from P60002 and transformed into SC5314 to integrate at the endogenous locus. Importantly, expression of the P60002 alleles in SC5314 increased transcript abundance to similar levels to those of the endogenous genes in P60002 (Fig. 2b). We suggest that elevated expression of the two genes in the recipient strains is due to polymorphisms that distinguish the gene promoters in P60002 and SC5314 (see Tables S5, S6). Thus, introduction of the P60002 allele of *CDR11* into SC5314 increased expression of this gene 46-fold, while introduction of the P60002 allele of *QDR1* increased total *QDR1* expression in SC5314 10-fold (Fig. 2b). Introduction of the P60002 *CDR11* allele reduced the zone of drug clearance by a small but not significant amount (*P*=0.21, Fig. S1). However, neither of the engineered SC5314 strains showed increased fluconazole resistance (*CDR11,* *P*=0.48; *QDR1,* *P*=0.96, Fig. 2c, d).

### Chromosome 4 trisomy contributes to increased drug resistance in P60002

**Fig. 3.**
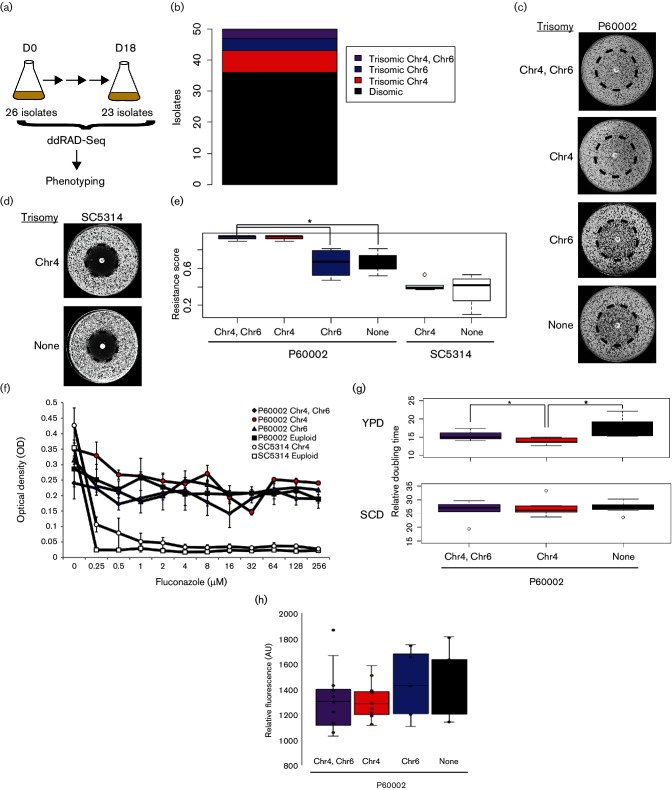
Chr4 trisomy contributes to increased fluconazole resistance in P60002. (a) Single colonies of P60002 were serially passaged for 18 days to induce loss of Chr4 and/or Chr6 trisomies. Forty-nine isolates were genotyped by ddRAD-Seq to assess chromosome copy number. (b) Schematic showing P60002-derived isolates that were disomic or trisomic for Chr4 and/or Chr6 combined for both D0 and D18 time points. (c) Analysis of fluconazole resistance in P60002-derived isolates that were disomic or trisomic for Chr4 and Chr6. The dashed circle indicates an area where cells grew more slowly when lacking the Chr4 trisomy. (d) Analysis of fluconazole resistance in SC5314 or in an aneuploid SC5314 derivative that harbours a Chr4 trisomy. (e) Fluconazole resistance in SC5314 and P60002 strains that were disomic or are trisomic for Chr4 and/or Chr6. Drug resistance for SC5314 isolates that were trisomic for Chr4 was collected from six independent aneuploid strains. (f) Optical density of broth microdilution assays across a range of fluconazole concentrations for P60002- and SC5314-derived strains that were disomic or trisomic for Chr4 or Chr6 (values determined after 24 h). Each strain–ploidy combination is represented by three biological replicates. (g) Doubling times for different karyotypic forms of P60002 when grown in liquid YPD or SCD medium. (h) Measurement of rhodamine 123 accumulation in P60002 or derivatives to assay MDR efflux pump activity. All plate images in the figure were taken after 2 days.

The P60002 isolate is trisomic for two chromosomes, Chr4 and Chr6 (Fig. 1), and we tested whether the presence of either of the supernumerary chromosomes contributes to elevated fluconazole resistance. P60002 was serially passaged in YPD medium for 18 days to induce spontaneous loss of one or both of the supernumerary chromosomes (Fig. 3a). Forty-nine isolates [including multiple day 0 (D0) and day 18 (D18) time points] were genotyped by double-digest restriction-site-associated DNA sequencing (ddRAD-Seq). Surprisingly, the majority (20/26) of sequenced isolates from the D0 samples were euploid (Fig. 3b), indicating that the original P60002 isolate consisted of cells in a mixture of different ploidy states. In fact, of the 15 isolates sequenced at both D0 and D18 points, 10 were euploid prior to passaging. Two passaged isolates were initially trisomic for both Chr4 and Chr6; one retained the trisomic chromosomes during passaging, while the other lost both supernumerary chromosomes. All three isolates that contained a single trisomic chromosome prior to passaging (two for Chr4 and one for Chr6) became disomic for these chromosomes by day 18. Together, these experiments identified P60002 populations with different copy numbers for Chr4 and Ch6, and established that isolates trended towards euploidy during *in vitro* passaging.

To assess the potential contributions of trisomic chromosomes to drug resistance in P60002, isolates encoding different disomic/trisomic combinations of Chr4 and Chr6 were grown in the presence of fluconazole. As expected based on our previous analysis [54], P60002 isolates that were trisomic for both chromosomes grew up uniformly in the presence of the fluconazole diffusion disk, demonstrating resistance even to high levels of the drug (Fig. 3c). In contrast, the standard laboratory strain SC5314 exhibited a large zone of clearance around the disk, which was indicative of drug susceptibility (Fig. 3d). Euploid derivatives of P60002 that had lost both Chr4 and Chr6 trisomies displayed intermediate levels of resistance, in which colonies still formed close to the fluconazole disk but with a reduced size (Fig. 3c). These growth characteristics are indicative of drug tolerance, reflecting a reduced ability of the drug to inhibit *C. albicans* growth [57]. Importantly, P60002 isolates that were trisomic for Chr4 exhibited similar fluconazole resistance levels to isolates that were trisomic for both Chr4 and Chr6 (*P*=1), whereas those that were trisomic for Chr6 showed resistance phenotypes that were similar to those of euploid P60002 cells (compared to trisomic Chr4 and Chr6: trisomic Chr6; *P*=0.016, disomic; *P*=0.015, Fig. 3c, e). These results implicate loci present on Chr4 as making key contributions to fluconazole resistance in P60002. We also compared the growth of euploid and aneuploid versions of P60002 in a fluconazole broth microdilution assay. Using this assay we found that karyotypic variants of P60002 displayed no noticeable growth differences across a range of fluconazole concentrations. This reflects how different resistance phenotypes are observed between *C. albicans* isolates grown on solid media versus liquid media (Fig. 3f) [57, 63–65].

The presence of supernumerary chromosomes in *C. albicans* isolates is often associated with reduced fitness relative to euploid isolates [66–68]. To determine the impact of supernumerary chromosomes on doubling times in P60002, the growth rates of aneuploid and euploid strains were measured in YPD and SCD media. Interestingly, strains carrying the Chr4 trisomy had shorter doubling times than strains that were euploid or strains that were trisomic for both Chr4 and Chr6 when cultured in YPD medium (Fig. 3g). In contrast, no significant differences in doubling times existed when strains were grown in SCD. These results indicate that the presence of a supernumerary chromosome does not always lead to a significant growth defect, at least when comparing doubling times during exponential growth under replete nutrient conditions.

To determine if Chr4 trisomy also increases fluconazole resistance in other *C. albicans* strain backgrounds, we took advantage of parasexual derivatives of SC5314 that are trisomic for this chromosome [68]. We utilized five derivatives of SC5314 that have a Chr4 trisomy and examined their growth in the presence of fluconazole diffusion disks. Notably, SC5314 strains that were trisomic for Chr4 did not exhibit elevated resistance relative to the euploid control (*P*=0.56, Figs 3d–f and S2). However, one strain that was trisomic for both ChrR and Chr4 did show increased fluconazole resistance relative to the control (*P*=0.02, Fig. S2). Thus, while gene dosage of loci on Chr4 plays a significant role in promoting fluconazole resistance in P60002, this effect is dependent on strain background and is not typically observed in the standard laboratory strain of *C. albicans*.

### Chr4 trisomy does not alter MDR efflux pump activity

To investigate if efflux pump activity provides elevated fluconazole resistance in P60002 isolates that are trisomic for Chr4, we performed Rh123 accumulation assays in strains with different combinations of Chr4 and Chr6. Removal of Rh123 from the cell requires activity of the multidrug-resistance (MDR) class of efflux pumps and correlates with azole resistance in *C. albicans* [69, 70]. Decreased Rh123 retention suggests hyperactive drug efflux that could also provide elevated drug resistance. All karyotypic variants of P60002 accumulated similar levels of Rh123 (Fig. 3h), suggesting that the expulsion of azoles by MDR efflux pumps does not contribute to elevated fluconazole resistance in P60002 harbouring a trisomic Chr4.

## Discussion

In this study, we investigated the genetic basis of fluconazole resistance in *C. albicans* isolate P60002. This isolate was of significant interest as it lacked any polymorphisms or gene expression patterns known to be associated with fluconazole resistance. Furthermore, we showed that increased expression of two genes, *CDR11* and *QDR1*, encoding putative drug efflux pumps had no effect on fluconazole resistance when transferred into the laboratory strain, SC5314. Instead, we established that a Chr4 trisomy makes a significant contribution to azole resistance in P60002, further extending the link between karyotypic change and drug resistance in fungal species.

Previous studies have demonstrated that many fluconazole-resistant isolates of *C. albicans* are aneuploid [15, 32, 45, 71, 72], although the precise relationship between specific aneuploid forms and drug resistance remains unclear. Fluconazole exposure was recently shown to induce aneuploid formation in *C. albicans* due to cell cycle aberrations [48], and a subset of these chromosomal alterations are known to provide fitness advantages in the presence of the drug. The most direct link between aneuploidy and drug resistance involves the formation of i5L aneuploids, which are commonly encountered in the clinic and increase fluconazole resistance through elevated *ERG11* and *TAC1* expression [15, 28]. More recent evidence suggests that trisomy of ChrR also contributes to increased azole resistance in *C. albicans* [46], and we similarly observed elevated fluconazole resistance in an SC5314-derived strain that was trisomic for both ChrR and Chr4 (Fig. S1). However, it is likely that many of the karyotypic changes observed in clinical isolates do not contribute directly to drug resistance. In addition, some aneuploid forms could represent a transitory state before cells achieve a more stable or less costly mechanism of resistance [45]. For example, studies in *Saccharomyces cerevisiae* have shown that supernumerary chromosomes can promote rapid adaptation to stressful environments, but more refined solutions then evolve with lower fitness costs than aneuploidy [33].

Fluconazole resistance appears to be multifactorial in isolate P60002, with Chr4 trisomy being one factor contributing to overall drug resistance. This isolate was recovered from the bloodstream of a patient and belongs to clade SA [54], but no other clinical information is available about its history. The genome-sequenced P60002 isolate was trisomic for both Chr4 and Chr6, and upon passaging in the laboratory both of the trisomic chromosomes were often lost, indicating that the supernumerary chromosomes had limited stability. This could be due to a fitness cost associated with aneuploidy, as an imbalanced karyotype has generally been associated with slower proliferation rates in a variety of species [73–75]. Notably, however, P60002 isolates that were trisomic for Chr4 grew marginally faster than euploid derivatives, at least when meassuring exponential growth in rich culture conditions. This is consistent with previous suggestions that *C. albicans* is well suited to tolerating aneuploidy [67], and with experiments in *S. cerevisiae* where some aneuploids were found to grow faster than isogenic euploids under a subset of conditions [15, 75, 76]. We also note that an increase in Chr4 copy number occurred in *C. albicans* isolate T118 when serially passaged in the presence of fluconazole, although the change in copy number was not shown to contribute to increased drug resistance [67].

Loss of the Chr4 trisomy reduced, but did not eliminate, the resistance profile observed in P60002. Thus, P60002 strains disomic for Chr4 were still highly tolerant to high concentrations of azoles, with extensive growth up to the fluconazole disk. This suggests that the resistance phenotype observed in the aneuploid P60002 strain is due to mechanisms that promote both tolerance and resistance to fluconazole. Derivatives that are disomic for Chr4 are no longer resistant but still exhibit considerable tolerance to the drug. Loss-of-function mutations for genes associated with *C. albicans* tolerance are not evident in P60002 [35, 38, 54], suggesting that additional mechanisms contribute to the underlying tolerance phenotype of euploid P60002 cells to fluconazole.

Analysis of fluconazole resistance in *C. albicans* has often focused on *ERG11* polymorphisms, copy number variation in key transcriptional regulators, or gene expression levels of drug efflux pumps. P60002 displayed elevated expression of two members of the ABC and MFS drug efflux transporter gene families, *CDR11* and *QDR1,* and ectopic expression of the P60002 alleles in SC5314 led to increased expression of these genes in this strain background. Elevated expression of the ectopic alleles in SC5314 is likely due to promoter polymorphisms that increase transcription, or to coding polymorphisms that alter RNA stability. Despite elevated expression of *CDR11* and *QDR1,* drug resistance was not enhanced in the fluconazole-susceptible SC5314 strain. This is consistent with a recent study that found no difference in azole susceptibility in *QDR1-*null mutants of SC5314 [77]. It is therefore apparent that as yet unknown mechanisms, in addition to the contribution of Chr4 trisomy, promote drug resistance in P60002. By extension, our results emphasize the difficulty of making genotype-to-phenotype connections, even in cases where strong candidate genes are predicted to underlie drug resistance.

Acquisition of drug resistance often occurs by incremental increases in tolerance to the drug. For example, previous studies established that activating mutations in *UPC2* and *TAC1* make additive contributions to drug resistance [25]. Resistance in P60002 also appears to be due to the combinatorial effects of multiple genetic alterations. The presence of the Chr4 trisomy confers an increase in fluconazole resistance, but other genetic changes must also contribute to overall drug resistance, as loss of this trisomy generated P60002 derivatives that still exhibited tolerance to the drug. Loss of the Chr4 trisomy occurs in the absence of selective pressure, demonstrating that it is readily dispensable in the absence of drug.

We also examined whether Chr4 trisomy provides increased fluconazole resistance to *C. albicans* isolates from other strain backgrounds. Along with P60002, two additional clinical isolates in the same collection were found to be trisomic for Chr4 (isolates 12C and P78042), but neither of these isolates exhibited elevated drug resistance [54]. To further address the relationship between Chr4 trisomy and drug resistance, we examined multiple derivatives of SC5314 that were trisomic for Chr4 [68]. None of the SC5314 strains that were trisomic for only Chr4 showed elevated fluconazole resistance, however, indicating that the influence of Chr4 trisomy is dependent on strain background. These findings establish that certain genetic changes have universal effects on drug resistance independent of strain background, but others vary considerably between isolates, and are consistent with observations in other systems where genetic background has been shown to have a major effect on mutant phenotypes [78, 79]. Future investigations will therefore need to carefully consider the role of genetic interactions in the emergence of drug resistance to generate a comprehensive understanding of this important problem.

## Supplementary Data

4396 Supplementary File 1
