# Supplementary material for: A chromosome 4 trisomy contributes to increased fluconazole resistance in a clinical isolate of Candida albicans
Source: Microbiology (Reading). 2017 Jun 22;163(6):856–65. doi: 10.1099/mic.0.000478 (PMC5737213; doi:10.1099/mic.0.000478)
Supplement: Supplementary File 1 [file mic-163-856-s001.pdf]

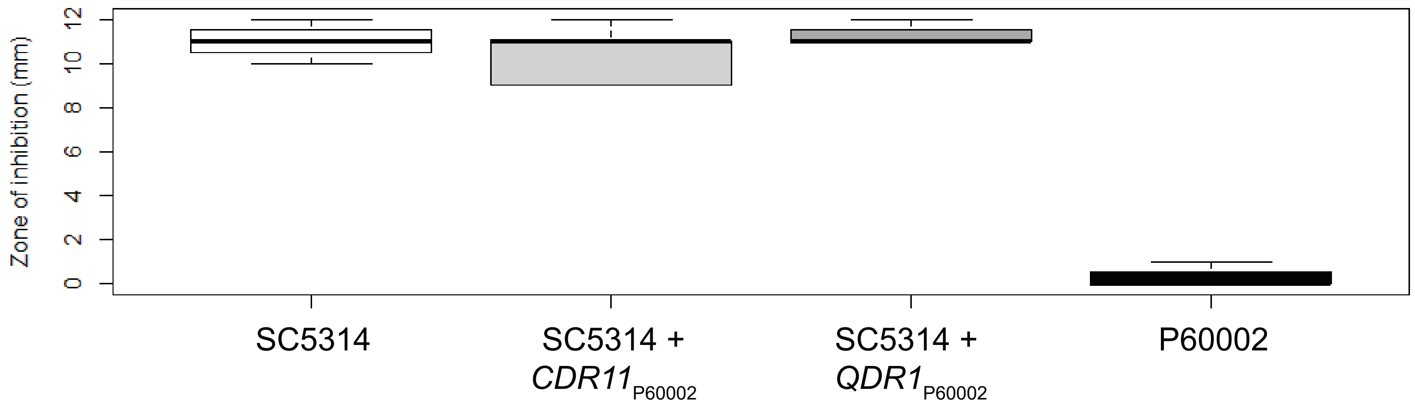

Zone of inhibition (mm)
0
2
4
6
8
10
12
SC5314
SC5314 + CDR11P60002
SC5314 + QDR1P60002
P60002

Figure S1

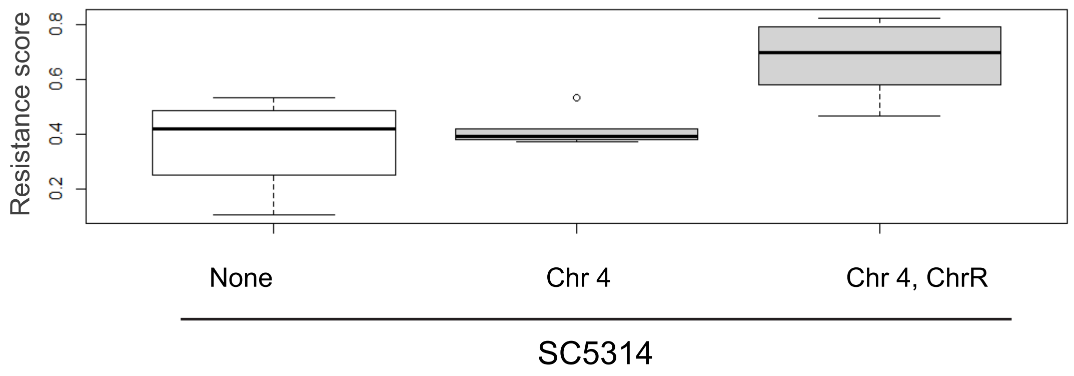

Figure S2

Table S1. Strains used in this study.

Strain(s)	Genotype	Strain Background
CAY682	WT (Trisomic Chr4, Chr6)	SC5314
CAY704/7401	WT	P60002
CAY6714/6715/6716	QDR1/qdr1::$QDR1_{P60002}$-SAT1	SC5314
CAY6897/6898	CDR11/cdr11::$CDR11_{P60002}$-SAT1	SC5314
CAY7402/7403/7405	Trisomic Chr4, Disomic Chr6	P60002
CAY7413/7414/7416	Disomic Chr4, Trisomic Chr6	P60002
CAY7417/7418/7419	Disomic Chr4, Disomic Chr6	P60002
CAY3102/3121	Trisomic Chr4	SC5314
CAY3105/3106	Trisomic ChrR, Trisomic Chr4	SC5314
CAY3108	Trisomic Chr4, Trisomic Chr7	SC5314

Table S2. Oligonucleotides used in this study.

Oligo	Name	Sequence
1	CDR11_Amplification_F	AACCGCGGCCCCTCCTCTCCAACAAATT
2	CDR11_Amplification_R	AAGCGGCCGCCCAACTTGCATTCAGATTCG
3	CDR11_Integrant	GTCAAACAATGGTGCTGCAG
4	SacII_5pr_QDR1_cloning	AACCGCGGGCCTGATTCTATACGACGAC
5	NotI_3pr_QDR1_cloning	AAGCGGCCGCCCATTCATCAACGGGTCTAC
6	QDR1_Integrant	ACAGAGTGGTGCATCTGCT
7	CDR11_qRTPCR_577_F	ATTGCTGTCAACACGTATGG
8	CDR11_qRTPCR_817_R	CATCGGCCATACGTTTAGCA
9	QDR1_qRTPCR_352_F	TGTGCCGTGTGCATTGCTAT
10	QDR1_qRTPCR_520_R	AACCAGAAACTGCACCAACC
11	Candida_ACT1_F_qRTPCR	TTGTACTCTTCTGGTAGAAC
12	Candida_ACT1_R_qRTPCR	GTTTGGTCAATACCAGCAGC

Table S3. Polymorphisms in the *CDR11* CDS.

Position	SC5314 allele	P60002 allele	Allele Frequency
Chr3: 849070	G	A	1.0
Chr3:849180	G	A	1.0
Chr3:849480	C	T	0.5
Chr3:849492	A	G	0.5
Chr3:849582	C	T	0.5
Chr3:849603	T	C	0.5
Chr3:849660	A	G	0.5
Chr3:849666	G	A	1.0
Chr3:849669	A	G	1.0
Chr3:849702	G	A	0.5
Chr3:849720	G	A	0.5
Chr3:849768	C	T	0.5
Chr3:849840	C	T	1.0
Chr3:849852	T	C	1.0
Chr3:849919	C	T	1.0
Chr3:850035	G	A	1.0
Chr3:850184	T	C	1.0
Chr3:850302	C	T	1.0
Chr3:850323	A	G	1.0
Chr3:850341	G	A	1.0
Chr3:850347	A	G	1.0
Chr3:850353	A	G	1.0
Chr3:850398	G	A	1.0
Chr3:850539	G	A	1.0
Chr3:850551	C	G	1.0
Chr3:850623	G	A	1.0
Chr3:850722	C	T	0.5
Chr3:850810	C	T	1.0
Chr3:850841	T	C	1.0
Chr3:850926	C	T	0.5
Chr3:850971	C	T	0.5
Chr3:850980	A	G	0.5
Chr3:851072	C	G	1.0
Chr3:851166	T	C	1.0
Chr3:851235	A	G	1.0
Chr3:851352	G	A	0.5
Chr3:851447	A	G	0.5
Chr3:851739	C	T	1.0
Chr3:852186	A	T	1.0
Chr3:852348	C	T	1.0
Chr3:852375	G	A	1.0
Chr3:852486	T	A	0.5
Chr3:852508	C	T	1.0
Chr3:852706	C	G	0.5
Chr3:852790	G	A	1.0
Chr3:853167	G	A	1.0
Chr3:853221	G	T	0.5
Chr3:853236	G	A	1.0

Table S4. Polymorphisms in *QDR1* CDS.

Position	SC5314 allele	P60002 allele	Allele Frequency
ChrR:941236	A	G	1.0
ChrR:941290	T	C	1.0
ChrR:941386	A	G	1.0
ChrR:941974	C	T	1.0
ChrR:942002	C	A	1.0
ChrR:942576	G	A	1.0
ChrR:942616	C	T	1.0
ChrR:942649	G	T	1.0

Table S5. Polymorphisms in the *CDR11* promoter.

Position	SC5314 allele	P60002 allele
Chr3: 853725	C	A
Chr3: 853713	G	T
Chr3: 853692	A	G
Chr3: 853686	G	A

Table S6. Polymorphisms in the *QDR1* promoter.

Position	SC5314 allele	P60002 allele
ChrR: 943319	A	G
ChrR: 943131	G	A
ChrR: 942999	G	A
ChrR: 942928	G	A
ChrR: 942901	A	G
ChrR: 942791	G	A
ChrR: 942754	T	G

SUPPLEMENTARY FIGURE LEGENDS

Figure S1. Zone of clearance is unaltered in SC5314 encoding *CDR11*$_{\mathbf{P60002}}$ or *QDR1*$_{\mathbf{P60002}}$ alleles. The zone of inhibition restricting 20% of growth was measured for each strain with 4-6 independent replicates.

Figure S2. An aneuploid derivative of SC5314 exhibits increased fluconazole resistance. A. Analysis of fluconazole resistance in SC5314, two SC5314-derived isolates trisomic for Chr4, and an SC5314-derived strain trisomic for ChrR and Chr4. Drug resistance for these isolates was collected from experiments with five to six independent replicates.

Table S1. Strains used in this study.

Table S2. Oligonucleotides used in this study.

Table S3. Polymorphisms in the *CDR11* CDS.

Table S4. Polymorphisms in the *QDR1* CDS.

Table S5. Polymorphisms in the *CDR11* promoter.

Table S6. Polymorphisms in the *QDR1* promoter.
